# Supplementary material for: Absence of Anti-Babesia microti antibody in commercial intravenous immunoglobulin (IVIG)
Source: PLoS Negl Trop Dis. 2024 Mar 14;18(3):e0012035. doi: 10.1371/journal.pntd.0012035 (PMC10965045; doi:10.1371/journal.pntd.0012035)
Supplement: S1 Table — Table with listed commercial IVIG samples used for ELISA and IFA testing, including collection date, product, lot number and ELISA results(s). (DOCX) [file pntd.0012035.s001.docx]

| **Sample Number** | **ELISA**  **OD450**  **1:32** | **ELISA**  **OD450**  **1:64** | **ELISA**  **OD450**  **1:128** | **Collection Date** | **Product** | **Lot Number** |
| --- | --- | --- | --- | --- | --- | --- |
| 1 |  | 0.111 | 0.097 | 7/1/2021 | Privigen | P100316995 |
| 2 |  | 0.111 | 0.086 | 7/1/2021 | Gammagard S/D | LE08W034AC |
| 3 |  | 0.125 | 0.12 | 7/1/2021 | Gammagard S/D | LE08W010AD |
| 4 |  | 0.095 | 0.075 | 7/1/2021 | Gammagard S/D | LE08V012AD |
| 5 |  | 0.096 | 0.086 | 7/13/2021 | Gammagard S/D | LE08W024AC |
| 6 | 0.191 | 0.174 | 0.129 | 7/13/2021 | Gammagard S/D | LE08W032AC |
| 7 |  |  |  | 7/13/2021 | Privigen | P100341284 |
| 8 | 0.214 | 0.16 | 0.127 | 8/3/2021 | Privigen | P100241853 |
| 9 |  | 0.111 | 0.104 | 8/3/2021 | Privigen | P100294932 |
| 10 | 0.255 | 0.233 | 0.13 | 8/9/2021 | Privigen | P100316992 |
| 11 |  |  |  | 8/9/2021 | Privigen | P100290691 |
| 12 |  |  |  | 8/3/2021 | Privigen | P100294932 |
| 13 |  | 0.115 | 0.093 | 8/12/2021 | Gammagard S/D | LE08W038AC |
| 14 |  | 0.096 | 0.089 | 8/26/2021 | Gammagard S/D | LE08X001AC |
| 15 |  | 0.117 | 0.1 | 9/10/2021 | Privigen | P100316994 |
| 16 | 0.478 | 0.382 | 0.206 | 9/10/2021 | Gamunex-C | B2GJC00343 |
| 17 |  | 0.156 | 0.105 | 9/23/2021 | Gammagard Liquid | LE12X034AB |
| 18 |  | 0.135 | 0.117 | 9/23/2021 | Gammagard Liquid | C21G036AAA |
| 19 |  | 0.108 | 0.095 | 9/23/2021 | Privigen | P100341671 |
| 20 |  | 0.114 | 0.094 | 9/23/2021 | Privigen | P100287719 |
| 21 |  |  |  | 9/23/2021 | Privigen | P100254258 |
| 22 | 0.231 | 0.194 | 0.159 | 10/8/2021 | Privigen | P100254261 |
| 23 | 0.317 | 0.189 | 0.109 | 10/8/2021 | Privigen | P100288426 |
| 24 | 0.307 | 0.187 | 0.131 | 10/8/2021 | Privigen | P100341288 |
| 25 |  | 0.092 | 0.082 | 10/8/2021 | Gammagard S/D | LE08X002AC |
| 26 | 0.273 | 0.146 | 0.115 | 7/1/2021 | Gammagard S/D | LE08W010AD |
| 27 | 0.293 | 0.166 | 0.121 | 11/1/2021 | Privigen | P100349926 |
| 28 | 0.259 | 0.169/0.096 | 0.101/0.1 | 11/15/2021 | Gammagard S/D | LE08X006AC |
| 29 |  | 0.141 | 0.093 | 11/1/2021 | Privigen | P100349926 |
| 30 | 0.261 | 0.134/0.102 | 0.131/0.096 | 12/23/2021 | Privigen | P100254262 |
| 31 |  | 0.171 | 0.14 | 12/23/2021 | Gamunex-C | B2GJC00553 |
| 32 |  | 0.227 | 0.177 | 1/6/2022 | Gammagard S/D | LE08W003AE |
| 33 |  | 0.102 | 0.097 | 1/6/2022 | Privigen | P100287724 |
| 34 |  | 0.12 | 0.096 | 1/6/2022 | Privigen | P100366291 |
| 35 |  | 0.114 | 0.088 | 1/6/2022 | Privigen | P100370200 |
| 36 |  |  |  | 1/6/2022 | Privigen | P100345683 |
| 37 |  | 0.128 | 0.154 | 1/6/2022 | Privigen | P100360211 |
| 38 |  |  |  | 1/6/2022 | Privigen | P100355519 |
| 39 |  |  |  | 1/6/2022 | Privigen | P100254263 |
| 40 |  | 0.29 | 0.159 | 1/6/2022 | Gammagard S/D | LE08X009AC |
| 41 |  | 0.12 | 0.091 | 1/31/2022 | Privigen | P100341290 |
| 42 |  | 0.118 | 0.091 | 2/21/2022 | Privigen | P100372895 |
| 43 |  |  |  | 2/21/2022 | Privigen | P100348228 |
| 44 | 0.362 | 0.426 | 0.133 | 2/21/2022 | Privigen | P100359889 |
| 45 | 0.238 | 0.147 | 0.144 | 4/8/2022 | Privigen | P100372893 |
| 46 | 0.223 | 0.164 | 0.13 | 4/13/2022 | Privigen | P100387313 |
| 47 | 0.237 | 0.162 | 0.136 | 4/13/2022 | Gammagard S/D | LE08X015AC |
| 48 | 0.217 | 0.140 | 0.099 | 4/20/2022 | Gammagard S/D | LE08X009AD |
| 49 |  | 0.117 | 0.088 | 4/20/2022 | Gammagard S/D | LE08W036AT |
| 50 |  |  |  | 4/26/2022 | Gammagard S/D | LE08X018AC |
| 51 | 0.253 | 0.193 | 0.132 | 4/26/2022 | Privigen | P100387309 |
| 52 |  | 0.086 | 0.067 | 4/26/2022 | Privigen | P100375300 |
| 53 | 0.282 | 0.307 | 0.122 | 4/26/2022 | Privigen | P100389255 |
| 54 | 0.287 | 0.180 | 0.136 | 4/26/2022 | Privigen | P100387320 |
| 55 | 0.279 | 0.139 | 0.138 | 5/18/2022 | Privigen | P100409786 |
| 56 | 0.268 | 0.188 | 0.165 | 5/18/2022 | Privigen | P100403997 |
| 57 | 0.342 | 0.187 | 0.116 | 5/19/2022 | Privigen | P100400600 |
| 58 | 0.126 | 0.235 | 0.113 | 5/19/2022 | Gammagard S/D | LE08X014AC |
| 59 | 0.169 | 0.129 | 0.11 | 5/19/2022 | Gammagard S/D | LE08X017AC |
| 60 | 0.251 | 0.153 | 0.142 | 5/19/2022 | Gammagard S/D | LE08X013AT |
| 61 | 0.212 | 0.116 | 0.101 | 5/19/2022 | Gammagard S/D | LE08X019AT |
