# Supplementary material for: Absence of Anti-Babesia microti antibody in commercial intravenous immunoglobulin (IVIG)
Source: PLoS Negl Trop Dis. 2024 Mar 14;18(3):e0012035. doi: 10.1371/journal.pntd.0012035 (PMC10965045; doi:10.1371/journal.pntd.0012035)
Supplement: S2 Table — Table with values of Babesia microti IgG, serum IgG, CD 20% and parasitemia %. (DOCX) [file pntd.0012035.s002.docx]

**S2 Table: Numerical values corresponding to Fig 1.**

| **Babesia microti IgG titers** | **Date** | **IgG titer** |
| --- | --- | --- |
|  | Jan 2018 | < 1 : 16 |
|  | Feb 2018 | 1 : 64 |
|  | Mar 2018 | 1 : 256 |
|  | Oct 2018 | 1 : 256 |
| **Serum IgG** |  | **Serum IgG (mg/dL)** |
|  | Dec 2017 | 337 |
|  | Jun 2018 | 998 |
|  | Aug 2018 | 848 |
|  | Sept 2018 | 609 |
|  | Oct 2018 | 531 |
| **CD20 %** |  | **CD20 %** |
|  | Jan 2018 | 0 |
|  | Aug 2018 | 10 |
| **Babesia microti % parasitemia** |  | **Parasitemia %** |
|  | Oct 2017 | 0.33 |
|  | Nov 2017 | 0.05 |
|  | Nov 2017 | 0 |
|  | Nov 2017 | 0 |
|  | Nov 2017 | 0.033 |
|  | Dec 2017 | 0.02 |
|  | Dec 2017 | 0.16 |
|  | Dec 2017 | 0.18 |
|  | Dec 2017 | 0.1 |
|  | Jan 2018 | 0.09 |
|  | Jan 2018 | 0.15 |
|  | Jan 2018 | 0.173 |
|  | Jan 2018 | 0.17 |
|  | Feb 2018 | 0.027 |
|  | Feb 2018 | 0.02 |
|  | Feb 2018 | 0.05 |
|  | Feb 2018 | 0.03 |
|  | Mar 2018 | 0.17 |
|  | Mar 2018 | 0.28 |
|  | Mar 2018 | 0.06 |
|  | Mar 2018 | 0.05 |
|  | Apr 2018 | 0.03 |
|  | Apr 2018 | 0 |
|  | Apr 2018 | 0 |
|  | Apr 2018 | 0 |
|  | May 2018 | 0 |
|  | Jun 2018 | 0 |
|  | Jun 2018 | 0 |
|  | Jul 2018 | 0 |
|  | Aug 2018 | 0 |
|  | Aug 2018 | 0 |
|  | Sep 2018 | 0 |
