# Supplementary material for: Absence of Anti-Babesia microti antibody in commercial intravenous immunoglobulin (IVIG)
Source: PLoS Negl Trop Dis. 2024 Mar 14;18(3):e0012035. doi: 10.1371/journal.pntd.0012035 (PMC10965045; doi:10.1371/journal.pntd.0012035)
Supplement: Attachment — Submitted filename: babesia_responsetoreviewers_12.16.23.docx [file pntd.0012035.s003.docx]

**Response to reviewers**

Dear Editors,

We sincerely appreciate your feedback and suggestions and have modified the manuscript accordingly. Please find responses to reviewer comments below.

Thank you for your time and effort in considering the publishing of our work.

**Methods:**

Reviewer 2

1. “*The study was designated appropriate and all analysis were performed correctly. I have one comment to clarify – why were only a portion of IVIG samples tested using ELISA, while 57 commercial samples were tested using IFA?”*

**Response:** Thank you for your question. We have tested an additional 28 IVIG samples that were available using ELISA and incorporated them into Figure #2. In total, we tested 52 commercial IVIG samples using ELISA.

**Summary and General Comments:**

Reviewer 2

2. “*The manuscript entitled "Absence of anti-Babesia microti antibody in commercial Intravenous immunoglobulin (IVIG)” deals with a very important aspect of the therapeutic effect of IVIG as adjunctive therapy in babesiosis treatment, especially in immunocompromised patients. Overall this is a well-conceived, relevant and rigorously implemented study, and I believe the results contained would further knowledge in this area. Therefore, I congratulate the authors on well-conducted research and valuable results. However, I have one comment to clarify – why were only a portion of IVIG samples tested using ELISA, while 57 commercial samples were tested using IFA? I think this short explanation should be added to the text.”*

**Response:** We have now tested all 52 commercial IVIG samples that were available using ELISA. We could not test 5 IVIG samples due to low sample volume.

Reviewer 3

“*This manuscript demonstrated the anti-Babesia microti antibody titers of commercial IVIG and can provide useful insight into the potential therapeutic efficacy of IVIG for babesiosis.
However, due to the several defects, it was determined that this manuscript was insufficient to publish.*

3. *1) Lack of information about a 66-year-old male donor

The donor information used for the data in Fig. 1 should be explained in the Materials and Methods section, not in the Results section.*

**Response:** As per these suggestions, the donor information was expanded and moved to the Material and Methods section.

4. *There was no information on whether informed consent was obtained from this donor.*

**Response:** A statement regarding informed consent was added to the manuscript (line 100).

5. *The information on the IVIG administered to this donor should be presented, along with a detailed list of the 57 samples tested in this study.*

**Response:** A table was added which lists details of all samples tested by IFA and ELISA (table S1). Unfortunately, the information about IVIG administered to the case patient is not available.

6. *Authors stated that this donor had B-cell lymphoma, but did not write when he received rituximab.*

**Response:** Details regarding rituximab administration were added to the manuscript (lines 90-92).

7. *The description of rituximab in the Discussion section should be presented in the Introduction section.*

**Response:** Some of the description of rituximab was moved to the Introduction section.

8. *In the Discussion section, the authors should discuss the results of the donor's serum IgG levels in relation to the history of rituximab administration, IVIG administration, etc.
Since clindamycin was administered at the same time as the IVIG in this study, the efficacy of IVIG should be discussed in considering of antibiotics, and should be compared with the results with antibiotics alone.*

**Response:** A paragraph was added in the Discussion section that describes the patient’s rise in antibody titers, and the concurrent use of antibiotics with IVIG (lines 228-247).

9. *In addition, it would be interesting to determine if anti-Babesia microti antibodies are present in the serum of this recovered donor.*

**Response:** Anti-*Babesia microti* antibodies were measured and were present in the case patient. The antibody titers are included in Figure 1 and will be highlighted in the ‘case report’ (line 103).

10. *2) Lack of detailed information about IVIG

More detailed information on all 57 IVIG samples, including name, lot, and collection site, should be presented in a supplemental table.*

**Response:** As mentioned in item 3 above, a new table has been created with the details of all IVIG samples used for ELISA and IFA (table S1). Unfortunately, despite our best efforts, we were unable to determine the collection site of samples.

11. *3) No IFA results

There were no figures or discussion of the IFA results for the 57 samples of IVIG.
For IFA, a comparison with the results of non-infected RBCs as controls is needed, but was not stated.*

**Response:** IFA data have been added to the Results section.

12. *4) ELISA problems

The product name of ELISA assay kit should be presented.
Even though the manufacturer's positive control was used, the PC results in Fig. 2 showed a large difference (> 1.0 OD) between the two replicates. It should be re-tested.*

**Response:** The product name of the ELISA assay was added to the Methods section (line 136). We tested 24 additional IVIG samples along with positive control from the manufacturer and patient positive control. The new data plots are indicated by the red data points below. The final figure shows these data, but they are not highlighted by the red color. No information is available from the manufacturer regarding the time of collection of the positive control sera which is a likely variable causing differences between the positive control antibody values. However, there is no statistical significance between the positive control values.
