# Supplementary material for: Absence of Anti-Babesia microti antibody in commercial intravenous immunoglobulin (IVIG)
Source: PLoS Negl Trop Dis. 2024 Mar 14;18(3):e0012035. doi: 10.1371/journal.pntd.0012035 (PMC10965045; doi:10.1371/journal.pntd.0012035)
Supplement: Attachment — Submitted filename: Response to reviewers 2.12.docx [file pntd.0012035.s004.docx]

**Response to reviewers**

Dear Editors,

We sincerely appreciate your feedback and suggestions and have modified the manuscript accordingly. Please find responses to reviewer comments below.

Thank you for your time and effort in considering the publishing of our work.

**Methods:**

Reviewer 3

1. “*Need to check the number of IVIG samples again in the full text. The number of IVIG lots appears to be 58 in Table S1, not 57 (line 108). In addition, was the data for 24 (or 28? according to the response to Reviewer 2) additional IVIG samples added?”*

**Response:** Thank you for noticing this discrepancy. After reviewing our samples, there were a total of 61, however only 57 were tested as some samples were from duplicate lots. With regards to samples added for ELISA, there were indeed 24 added, not 28.

**Results:**

Reviewer 3

2. *“In the Methods section, ELISA was explained after IFA, so the same order should be used in the Results section (lines 159-176).”*

**Response:** This was edited, as per the recommendation (lines 159-176).

**Editorial and Data Presentation Modifications:**

Reviewer 3

*3. “Regarding the duplicate statement that rituximab depletes B cells in lines 73 and 77, delete one of them.”*

**Response:** As per the suggestion, one line stating the depletion of B cells was deleted in line 73.

*4. “The differences between A, B, and C attached to the sample numbers in Table S1 should be explained in the legend. Alternatively, they should be described as one lot with one sample number.”*

**Response:** As there is no meaningful difference between samples A, B and C, they were combined together in Table S1.

*5. “If possible, the results of IFA and the ELISA OD450 value used for Fig 2 should be added in Table S1. This would make it easier for the reader to understand which samples were tested in IFA and ELISA.”*

**Response:** Thank you for this recommendation. The ELISA OD450 values were added to table S1. Dilutions to 1:32, 1:64, and 1:128 were performed originally.  After reviewer suggestions, we included other IVIG samples that we had.  To accommodate all remaining specimen, we decided to only perform titration at 1:64 and 1:128. Some of the IVIG samples were mistakenly run again, therefore, they have another value from second run, which can be found in table S1.

Regarding IFA results, 57 samples were tested at four concentrations of 200 µg/ml, 100 µg/ml, 50 µg/ml, and 25 µg/ml. All were negative. There is no value to give for negative IFA results, therefore these cannot be added to table S1.
